# Perceptions of Health Risks from Hot Weather, and Coping Behaviors among Ethnic Minority Groups in Mountain Areas of China: A Case Study in the Tujia and Miao Autonomous Prefecture

**DOI:** 10.3390/ijerph15112498

**Published:** 2018-11-08

**Authors:** Hui Ye, Juan Ma, Yang Wu, Ying Zhang

**Affiliations:** 1 School of Public Management, South-Central University for Nationalities, Wuhan 430074, China; 2016110094@mail.scuec.edu.cn (J.M.); 2018110095@mail.scuec.edu.cn (Y.W.); 2 School of Public Health, University of Sydney, Sydney, NSW 2006, Australia; ying.zhang@sydney.edu.au

**Keywords:** extreme high temperature, health risk, perception, coping behavior, ethnic minorities, China

## Abstract

Limited research focuses on risk perceptions of hot weather among ethnic minority groups in remote mountain areas of China. Adopting a multi-stage sampling method, this study received completed questionnaires from 643 participates in Enshi Tujia and Miao Autonomous Prefecture of China in 2017 and 2018. We used multivariate logistic regression models to explore the factors affecting risk perceptions and coping behaviors with regards to hot weather. Results showed that despite a relatively high level of risk perception, the study population in the mountain areas of China had a very low level of preparedness in responding to the risks from heat, and a lack of professional health knowledge in general. In particular, 61.3% (95% CI: 57.1%−5.6%) of the participants felt increasing temperatures in recent years, 73.2% (95% CI: 69.3%−7.0%) thought extreme high temperatures would be a health threat, and 61.3% (95% CI: 57.1%−5.4%) reported physical discomfort during hot weather. However, only 12% (95% CI: 9.5%−4.5%) had the information or knowledge to stay healthy during the extreme high temperatures, and only 24.2% had (95% CI: 20.8%−7.6%) preparation. The logistic regression models suggested that ethnic group, health status, marital status, gender, and employment could affect their perceptions, which could significantly affect the adoption of coping behaviors. In conclusion, our findings have significant implications for developing policies and health education and promotion programs for ethnic minorities in remote regions to maintain good health during hot weather.

## 1. Introduction

Global climate change is bringing more and more hot weather across the world, which has threatened human health [1,2,3]. According to the fifth Assessment Report of the Intergovernmental Panel on Climate Change (IPCC), the global surface temperature has continued to rise since 1880; the average global temperature has increased by 0.85 °C in an accelerated manner [4]. In the summer of 2018, many countries in Asia, Europe, and North America experienced extreme heat events. The Chinese Meteorological Department announced that the summer of 2018 was the hottest since 1961. One of the most important and direct impacts of climate change is the health effect from very high temperatures [4]. High temperatures could push the human body’s core temperature regulation system to an overload state, leading to injured organs and tissues that are unable to function normally. The study on heat-related mortality in 66 counties in China from 2006 to 2011 reported 5% excess mortality due to extreme high temperatures [5]. Studies in other regions of China have also found increased numbers of hospital visits and ambulance callouts related to extreme heat events [6,7,8].

Studies have shown that health risk perception is an important psychological factor to predict adaptive behaviors. Evidence shows that those with a higher level of risk perception could be more likely to accept and respond to warning information, compared with those who do not perceive the risks [9,10,11]. Previous research suggests that the factors affecting public perception generally include external environment (such as meteorological conditions, communal facilities, and policies), individual characteristics (such as age, gender, race or ethnicity, and health status), and socioeconomic status (such as education level, occupation, marriage, and income). However, the effect of each factor has not been fully understood. Most vulnerable groups that were chosen as the study subjects of previous studies focused on older people, children, and patients with cardiovascular and cerebrovascular diseases, but there was a distinct lack of studies on race, immigrants, and ethnic minority groups. Research from the United States and Australia has found that social and economic disparities, living conditions, language barriers, and occupational exposure are among the factors contributing to heat-susceptibility among these groups [12]. Evidence shows that lower socioeconomic and ethnic minority groups were more likely to live in warmer suburbs with a greater exposure to heat stress in Philadelphia, USA [13,14]. Studies in Australia have found that older people of migrant background are at higher risks from extreme high temperatures, and reported more than one third of patients hospitalized with direct heat-related illnesses during extreme heat wave were non-Australian born [15,16].

There are very few studies examining perceptions of health risks from extreme high temperatures and coping behaviors among ethnic minority groups in mountain areas of China. One of the potential reasons for this may be the misperception that there may be no extreme heat in the areas. However, temperatures are getting higher in the mountain areas. For instance, there were 14 continuous days in which maximum temperature reached 38 °C in the Enshi Tujia and Miao Autonomous Prefecture of Hubei Province in 2018, which is in the Wuling Mountain region. In addition, the ethnic minorities who live in China’s remote areas have poor health infrastructures, greater poverty, and particularly, cultural diversity. Compared to Han people (the ethnic majority in China), minority residents have shorter life expectancies and utilize medical services less frequently [17,18].

The study area in the Wuling mountain region has typically been cool in summers in the past, with only rare extreme heat events. Having lived in the study area for thousands of years, the ethnic minority groups may not have the awareness and skills to respond to a warming climate. In particular, we do not know whether their traditional way of dealing with changes in weather is still effective in reducing associated adverse health outcomes. Given more frequent and severe heat events are projected due to climate change, our study is of particular significance in order to achieve better adaptation among the community. To address the urgent need to pay more attention to the health risks associated with hot weather among ethnic minority residents, our study aimed to provide more evidence to support local policy and decision making to build community resilience. 

## 2. Materials and Methods

Cross sectional surveys were conducted in Enshi Tujia and Miao Autonomous Prefecture of Hubei Province (Enshi Prefecture for short) from July to August in 2017 and 2018. We used a multi-stage sampling method to randomly select local residents. After the descriptive analysis, multivariate logistic regression models were developed to explore the factors affecting the risk perceptions and coping behaviors with regards to hot weather. All analyses were performed by STATA v14 with a significant level of 0.05.

### 2.1. Study Population

Enshi Prefecture (108°23′12″ E−10°38′08″ E, 29°07′10″ N−1°24′13″ N) is located in the southwest of Hubei Province, and in the Wuling Mountain region. The Enshi Prefecture Government was first established on 19 August 1983, and it is the only autonomous prefecture of ethnic minority in Hubei Province where the main ethnic groups are Tujia and Miao. Enshi is the home of diverse native plants that are sensitive to global climate change. It has a subtropical monsoon mountainous humid climate, with an average annual temperature of 16.2 °C and average annual precipitation of 1600 mm [19]. Summers (July and August) are getting hotter in Enshi. There were 170 days having a maximum daily temperature higher than 35 °C between 2011 and 2018, showing a significant increasing trend [20]. 

### 2.2. Survey and Sampling

A multi-stage sampling method was adopted. There are five counties in Enshi Prefecture, i.e., Enshi City, Xuan’en County, Hefeng County, Laifeng County, and Xianfeng County, which are all in mountainous areas. Firstly, two towns were selected from each county by simple random sampling. Secondly, stratified sampling was used to select one richer or poorer village from each selected township according to GDP per capita of all villages to ensure each county has one richer village and one poorer village. Finally, clustered sampling was used to select all adult household members in the ten villages, if the main householder was an ethnic minority resident. Thus, the sample covers residents from ten villages of ten townships in the five counties in Enshi prefecture. 

We administered 680 questionnaires to participating households and 643 questionnaires were completed, a response rate of 94.6%. The questions of the questionnaire covered household demographic and economic situations, health status, and the utilization of medical services, as well as their perceptions of health risks from extreme high temperatures and coping behaviors.

### 2.3. Measurement of Variables

Perceptions of warming temperatures and associated health risks were measured by the responses to the following questions, “Do you think the weather is getting warmer in recent years in your village?”, ”Do you think that high temperatures are a threat to human health?”, and “Do you feel that higher temperatures have caused physical discomfort of yourself?”. Questions on coping behaviors included “Do you have any message about how to maintain good health during extreme hot weather?”, ”Do you have any preparation at home (e.g., extra food and water) for extreme heat events?”, ”Do you take any of the following actions to respond to extreme heat events : drinking extra water, going somewhere cooler, staying indoors, using fan or air conditioner, or wearing lighter clothes?”, and” Whether you would ask for help if you were affected by an extreme heat event?”. The answer “yes” to each of these questions was assigned a value of 1; otherwise, 0 was assigned. 

Questions about health status were based on similar questions in the Fifth National Health Services Survey in China, including “Do you think you are healthy?”, “Do you have a chronic disease?”, “Do you suffer from a critical disease (examples given)?”, and “Have you been sick for the last two weeks?” The answer “yes” to each of these questions was assigned a value of 1; otherwise 0 was assigned. Chronic diseases included chronic infectious diseases (such as tuberculosis) and chronic non-infectious diseases (such as coronary heart disease and hypertension). Critical diseases referred to diseases that seriously affect normal work and life of individuals and their families, and often incur high medical costs, such as malignant tumors, severe cardiovascular and cerebrovascular diseases, and severe Parkinson’s disease [21].

### 2.4. Statistical Anlayses

Descriptive analysis of the proportions of perceptions and coping behaviors was given, and estimation of the situation in the whole study population was presented by the 95% Confidence Intervals (Cis). Three separate multivariate logistic models were developed to examine potential factors that could affect the perceptions of warming temperatures, health risks, and physical discomfort, and four separate models were developed to assess whether the perceptions could affect the adoption of different coping behaviors. Odds Ratios (ORs) and 95% Confidence Intervals (CIs) were presented.

### 2.5. Ethics Approval

The study was approved by the Ethics Committee of South-Central University for Nationalities in China in 2017 (2017-SCUEC-MEC-008).

## 3. Results

### 3.1. Descriptive Analyses

The majority (79.5%) of the participants were Tujia ethnic minorities. It was found that 91.1% of the participants were employed at the time of interview, 56.6% were male, and 24.7% were 65 years and older, with a mean age of 55 years. Only 7.8% had ever received education beyond high school, and 85.9% had a spouse. Also, 24.3% participants reported generally poor health status, with 57.6% and 11.1% having a chronic or critical disease. The two-week morbidity rate was 8.6% (Table 1).

The majority of the participants (61.3%) had perceived warming temperatures and recognized the associated health risks (73.2%). It was found that 61.3% participants reported physical discomforts during hot weather. However, only one quarter had preparation at home (e.g., extra food and water). In addition, there was a clear lack of health knowledge, with only 12% of people having information about how to maintain good health during hot weather. Nonetheless, most of them could take action to respond to hot weather, and 62.5% of the participants would ask for help if affected by extreme hot weather (Table 2).

**Figure 1:**
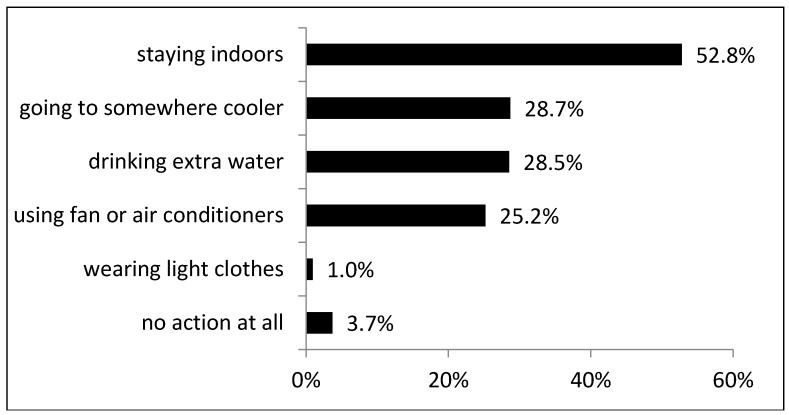
Choices of participants’ coping behavior during hot weather.

In terms of various types of coping behaviors, Figure 1 shows that 52.8% would choose to stay indoors, 28.7% would go to somewhere cooler, 28.5% would drink extra water, 25.2% would use a fan or air conditioner, and 1% would wear light-colored clothes.

### 3.2. Factors that could Affect the Perceptions and Behaviours

The logistic models indicated that ethnic group, health status, marital status, gender, and employment can significantly influence perceptions. Those who are Tujia ethnic minority, with poorer health status, or without a spouse, were more likely to be at risk. Those who were unemployed were more likely to suffer physical discomfort during hot weather (Table 3).

We further identified that these perceptions could significantly affect the coping behaviors. Those who perceived warming temperatures were more likely to have relevant health information (OR = 2.6) and more likely to prepare for hot weather (OR = 1.8). Those who perceived associated health risks were more likely to prepare before hot weather (OR = 2.2) and ask for help (OR = 1.9). However, those perceived physical discomforts during heat were less likely to take adaptive actions (OR = 0.2) (Table 4).

## 4. Discussion

Our study has examined risk perception of hot weather and coping behaviors among ethnic minority groups in mountain areas of China. The results indicate that more than half of the participants have perceived warming temperatures and are aware of the associated health risks. However, it is notable that the knowledge and preparation for adaptation are very low among the study population. We have detected that ethnic minority, health status, marital status, gender, and employment could affect their perception, which could subsequently affect their coping behavior. 

We have found a relatively high level of perceived warming temperatures and associated health risks in the study area, consistent with the records from meteorological bureau and findings from other studies. Based on the weather data of Enshi Tujia and Miao Autonomous Prefecture of Hubei Province, the number of hot days (>35 °C) and the duration of heat waves are both increasing, which is in line with the background of global warming. The 2018 heat wave lasted for 14 days, with a highest temperature of 38 °C. There are some international studies suggesting that the general public perception of the health risks from heat wave is low [22,23], because people tend to forget or adapt to the effects of extreme high temperatures [24]. The reason why this study population has a relatively higher proportion of risk perception related to heat cannot be determined. In addition to having more hot days in the region, this finding may be related to the cultural and traditional life styles of farmers that have a close connection and higher sensitivity to changes in the environment and climate, as well as poor access to quality health care. 

It is noted that only 12% had received information regarding how to stay healthy during extreme high temperatures. This may be because the ability among the ethnic minority to understand health information is lagging significantly behind that of Han, or the challenges in spreading information to remote regions [25]. A study in Italy also reported a high level of risk perception of extreme high temperatures, but a lack of needed knowledge to adapt [26]. However, this does not mean there is no adaption at all in the study area. In fact, although preparedness at home before hot days is very low in the study population, almost all of them would choose adaptive behaviors in response to heat. Although we did not expect local residents to have sufficient adaptive behavior based on their past experience, i.e., given relatively cooler temperatures in the mountain region in the past, there might be some traditional ways that have been passed onto them for responding to hot weather, such as going to a shared cooler place in the community and drinking extra water. We also do not know when the adoption of coping behaviors started in the community, and whether it is due to more frequent extreme heat events in the region recently. It is acknowledged that choices of adaptive behavior could vary greatly across regions and populations. More than half of the study population would choose to stay indoors, but less than 30% would drink extra water or use a fan [25]. In other studies, drinking more water is the most frequently-adopted adaptive behavior [27,28]. The lower frequencies of drinking more water and using fan may reflect local conditions of limited access to tap water and electricity in households, which should be taken into account in developing adaptive strategies. 

We have also found that a higher level of perception can significantly increase the adoption of coping behaviors, which is consistent with other studies. It shows that people’s perceptions of health risks will stimulate them to proactively avoid disaster from hot weather [26]. However, even if people feel unwell during heat, they may not be able to adopt appropriate adaptive behaviors. It is acknowledged that there are always limitations in interpreting the relationship between perceptions and adoption of coping behaviors in cross-sectional studies. Further longitudinal studies will be conducted based on established cohorts of ethnic minorities in the study region.

Being part of the Tujia ethnic minority may give rise to a higher level of perception of warming temperatures. Several international studies have reported that individuals from ethnic minorities are at increased risk of heat-related illness [12]. In our study, 57.6% of the participants have a diagnosed chronic disease, and Tujia ethnic minorities reported a higher proportion of physical discomfort during hot weather. A study in Australia has reported that minorities and immigrants were more susceptible to extreme heat and climate change [12]. A study in Hainan Province of China also reported a higher medical need among ethnic minority residents [25]. 

In addition to ethnic groups, we have identified other factors that could affect perception, including health status, employment, marital status, and gender. Compared with participants who are in good health, those with a diagnosed disease may have a stronger perception of health risks. These people are more vulnerable to extreme heat [29,30,31]. In addition, according to the National Bureau of Statistics of China, the average life expectancies of residents in eight provinces with large ethnic minority populations are all lower than the national average, but the average hospitalization rates among ethnic minorities were also lower [17,18]. Policies should be developed to attract more medical professionals to remote regions which face greater health risks from increasing temperatures. 

Employment status and marital status could affect risk perception. This finding is reasonable, as we have generally older participants, and those who are unemployed are mainly older people or patients without working abilities. Those without a spouse are usually widowed elderly in the villages. These people are the most vulnerable to the health risks of extreme high temperatures, and would be more likely to report discomfort during hot weather. Policies and health promotion programs should focus on these people in order to reduce associated disease burdens.

In our study, males are more likely to feel physical discomfort during heat. This is consistent with other studies [32,33]. However, previous findings are not consistent. For example, women expressed significantly greater awareness and sense of the perceived impacts of climate change compared to men in Alberta, Canada [34]. This may be due to different socio-economic status and roles in traditional life styles of the study population, as men are always the main householder who earns the income by doing more work. In addition, as the decision-makers of the family, men have more access to resources and education than women. Therefore, they may have a better understanding of the changing environment and associated impacts. This suggests that to address gender inequity in the study region, empowering women would be very critical given their role in the community, including looking after children and older people in households. 

## 5. Conclusions

Our study has analyzed the perception of health risks from hot weather, and coping behaviors among ethnic minority residents in mountain regions of China. Despite a relatively high level of risk perception, the study population has a very low level of knowledge, information, and resources to respond to the risks. We have also found that ethnic group, health status, marital status, gender, and employment could affect their perception, which can significantly influence coping behaviors. Our findings provide significant implications for developing policies and health education and promotion programs for ethnic minorities in remote regions with the aim of maintaining good health during hot weather.

## Figures and Tables

**Table 1:** Main characteristics of the participants.

	Proportion	95% CI
Tujia ethnic minority	79.5%	(76.3%, 82.6%)
Having employment	91.1%	(88.9%, 93.3%)
Male	56.6%	(52.8%, 60.5%)
Age 65 years and older	24.7%	(21.3%, 28.0%)
Education beyond high school	7.8%	(5.7%, 9.9%)
Married	85.9%	(83.2%, 88.6%)
Health status		
Self-reported a poor health status	24.3%	(21.0%, 27.7%)
Having a chronic disease	57.6%	(53.8%, 61.5%)
Having a critical disease	11.1%	(8.6%, 13.7%)
Two-week morbidity rate	8.6%	(6.5%, 10.8%)

**Table 2:** Perceptions and coping behavior among the total study population.

	Proportion	95% CI
Perceptions		
Perception of warming temperature	61.3%	(57.1%, 65.6%)
Perception of associated health risks	73.2%	(69.3%, 77.0%)
Perceived physical discomfort during hot weather	61.3%	(57.1%, 65.4%)
Coping behaviors		
Having relative health information	12.0%	(9.5%, 14.5%)
Having preparation before hot weather	24.2%	(20.8%, 27.6%)
Taking adaptive actions during hot weather	92.4%	(90.3%, 94.5%)
Asking for help	62.5%	(58.5%, 66.6%)

**Table 3:** Logistic regression models for the perceptions with ORs of the factors.

	Odds Ratio	Robust Std. Err.	*p* Value	95% CI
**Model 1—Perception of warming temperatures**				
Two-week morbidity rate	2.269	0.792	0.019	(1.144, 4.497)
Married	0.563	0.164	0.049	(0.318, 0.997)
Tujia ethnic minority	2.615	0.624	<0.001	(1.638, 4.175)
**Model 2—Perception of associated health risks**				
Having a chronic disease	2.023	0.428	0.001	(1.336, 3.063)
Having a critical disease	2.189	0.843	0.042	(1.029, 4.658)
**Model 3—Perceived physical discomforts during hot weather**				
Two-week morbidity rate	2.416	0.941	0.024	(1.126, 5.185)
Having employment	0.396	0.148	0.013	(0.191, 0.822)
Male	1.795	0.329	0.001	(1.253, 2.571)
Tujia ethnic minority	1.617	0.357	0.029	(1.049, 2.491)

**Table 4:** Logistic regression models for the coping behaviors with ORs of the factors.

	Odds Ratio	Robust Std. Err.	*p* Value	95%CI
**Model 1—Receiving health information**				
Perception of warming temperatures	2.593	0.879	0.005	(1.334, 5.038)
**Model 2—Preparing before hot weather**				
Perception of warming temperatures	1.790	0.490	0.033	(1.047, 3.063)
Perception of health risks	2.194	0.723	0.017	(1.150, 4.184)
**Model 3—Taking adaptive actions during hot weather**				
Perceived physical discomforts during heat	0.208	0.101	0.001	(0.080, 0.540)
**Model 4—Asking for help**				
Perception of health risks	1.891	0.409	0.003	(1.237, 2.891)
